# Socioeconomic inequality in recovery from poor physical and mental health in mid-life and early old age: prospective Whitehall II cohort study

**DOI:** 10.1136/jech-2017-209584

**Published:** 2018-02-08

**Authors:** Akihiro Tanaka, Martin J Shipley, Catherine A Welch, Nora E Groce, Michael G Marmot, Mika Kivimaki, Archana Singh-Manoux, Eric J Brunner

**Affiliations:** 1 Department of Epidemiology and Public Health, University College London, London, UK; 2 Department of General Medicine and Health Science, Nippon Medical School, Tokyo, Japan; 3 INSERM U1018, Université Paris-Saclay, Villejuif, France

**Keywords:** mental health, ageing, inequalities, cohort studies, functioning and disability

## Abstract

**Background:**

Few studies have examined the influence of socioeconomic status on recovery from poor physical and mental health.

**Methods:**

Prospective study with four consecutive periods of follow-up (1991–2011) of 7564 civil servants (2228 women) recruited while working in London. Health was measured by the Short-Form 36 questionnaire physical and mental component scores assessed at beginning and end of each of four rounds. Poor health was defined by a score in the lowest 20% of the age–sex-specific distribution. Recovery was defined as changing from a low score at the beginning to a normal score at the end of the round. The analysis took account of retirement status, health behaviours, body mass index and prevalent chronic disease.

**Results:**

Of 24 001 person-observations in the age range 39–83, a total of 8105 identified poor physical or mental health. Lower grade of employment was strongly associated with slower recovery from poor physical health (OR 0.73 (95% CI 0.59 to 0.91); trend P=0.002) in age, sex and ethnicity-adjusted analyses. The association was halved after further adjustment for health behaviours, adiposity, systolic blood pressure (SBP) and serum cholesterol (OR 0.85 (0.68 to 1.07)). In contrast, slower recovery from poor mental health was associated robustly with low employment grade even after multiple adjustment (OR 0.74 (0.59 to 0.93); trend P=0.02).

**Conclusions:**

Socioeconomic inequalities in recovery from poor physical health were explained to a considerable extent by health behaviours, adiposity, SBP and serum cholesterol. These risk factors explained only part of the gradient in recovery for poor mental health.

## Introduction

Socioeconomic inequalities in health are thought about primarily in terms of gradients in incident disease and mortality.^1^ An equally important dimension of health inequality relates to perceived poor mental and physical health,^2–4^ which may have a greater impact on quality of life than living with a chronic disease.^5^ Prevalence of poor health and corresponding functional limitations is determined by the rate of remission as well as the rate of incidence. As a result, a socioeconomic gradient in recovery from low health-related functional states is sufficient to produce inequality in the prevalence of suboptimal health, even if incidence does not differ by socioeconomic status.

It is to be expected that individuals with relatively robust financial and psychosocial resources are more resilient in their response to incident disease and loss of function. Hospital and register-based studies show faster functional improvements and return to work among higher compared with lower socioeconomic groups of psychiatric patients.^6 7^ A primary-care based study found that higher education level was associated with faster recovery from back pain.^8^ After stroke, patients discharged to home in less deprived areas were less likely to become dependent or to die.^9^ However, recovery from poor self-perceived health in the general population has been little studied from a health inequality perspective, and existing findings are conflicting.^10–13^

We examined socioeconomic inequalities in recovery from poor self-reported mental and physical health over 23 years according to employment grade defined by occupation and analysed the role that chronic disease, health behaviours, body mass index (BMI) and other physiological risk factors might play in producing the recovery dimension of health inequality.

## Methods

The Whitehall II cohort study was established in 1985, based on 10 308 civil servants (3413 women) aged 35–55 years recruited from 20 offices in London. The response rate was 73%.^14^ Data collections involving completion of a self-administered questionnaire and a clinical examination were made approximately every five years.^14^ We analysed data across four 5-year periods between 1991 and 2013 (table 1). Participants were followed into retirement, regardless of their employment history, and eligible to be included in a period if they completed the Short-Form 36 (SF-36) questionnaire at beginning and end of the period. All participants provided informed consent.

**Table 1:** Number of participants available in each observation period and the numbers with poor physical and mental health at the beginning and recovery at the end of each period

	Period 1	Period 2	Period 3	Period 4
Start of period	Phase III (1991–1994)	Phase V (1997–1999)	Phase VII (2002–2004)	Phase IX (2007–2009)
End of period	Phase V (1997–1999)	Phase VII (2002–2004)	Phase IX (2007–2009)	Phase XI (2012–2013)
Health scores at both phases, N	6605	5916	5840	5640
Poor physical health at start of period, N (%)	1186 (18.0)	1145 (19.4)	1302 (22.3)	1066 (18.9)
Recovered physical health at end of period, N (%)	580 (48.9)	504 (44.0)	649 (49.9)	492 (46.2)
Poor mental health at start of period, N (%)	1096 (16.6)	1201 (20.3)	1217 (20.8)	1070 (19.0)
Recovered mental health at end of period, N (%)	516 (47.1)	597 (49.7)	630 (51.8)	460 (43.0)

### Outcome

The SF-36 was administered on five occasions. The 36-item questionnaire has eight scales: physical functioning, role limitations due to physical problems, bodily pain, general health, vitality, social functioning, role limitations due to emotional problems and general mental health.^15 16^ The scales can be summarised into mental component score (MCS) and physical component score (PCS) based on factor analysis to produce two scores scaled from 0 to 100 (high score indicating good health) with mean 50 and SD 10, using the US population as reference.^17 18^ MCS and PCS differ by age and sex, and we used data from all five phases to generate age–sex-specific cut-points in order to define poor health as the lowest 20% of the distribution (online supplementary tables S1 and S2). Recovery from a low MCS and PCS was defined as improvement from a low to a normal score, that is, above the cut-point for age and sex, between the beginning and the end of each round.

10.1136/jech-2017-209584.supp1 Supplementary file 1

### Variables

The socioeconomic status of each participant was verified in each period, based on last known (current or most recent) civil service employment grade. Employment grade was assigned by combining official occupational grade into three categories: high (administrative), intermediate (professional or executive) and low (clerical or support officer). Employment grade classifies the cohort by salary, job characteristics such as control over work content and opportunity for use of skills, and social status.^14^

Health-related behaviours (smoking, alcohol intake and physical exercise) and health status (BMI, long-standing illness, coronary heart disease (CHD), stroke, diabetes mellitus, systolic blood pressure (SBP) and serum cholesterol) were derived from self-reports and clinic data at the beginning of each period.

### Statistical methods

The analytic sample was defined as person-observations with poor physical or mental health at the beginning and a valid SF-36 score at the end of any of the four periods. We summarised risk factors at the start of each of the four periods. We imputed missing risk factor data using multiple imputation which replaces missing values with randomly selected draws from the missing data distribution conditional on the observed data, specified by an imputation model, creating multiple imputed datasets.^19^ Multiple imputation accounts for uncertainty due to missing data and obtains unbiased estimates and SE if the missing at random assumption is plausible, that is, the reason for the missing data is associated with observed, but not unobserved, data.^19^ The imputation model included the risk factors described earlier, MCS and PCS and the following auxiliary variables which were associated with at least one of the risk factors with missing data.^20^ The risk factors were total cholesterol, SBP, fruit and vegetable consumption, chest pain, angina, high general health questionnaire score and receiving antihypertensive drug treatment at the beginning of the period and family history of angina, heart attack, stroke, high blood pressure or diabetes. We used 10 cycles to impute missing values in each imputed dataset and generated 20 imputed datasets which were analysed separately and the results combined using logistic regression models for recovery from poor physical and mental health, adjusted for age and sex, for each period separately. Socioeconomic gradients in recovery did not differ across periods; therefore, the four periods of observation were combined. We fitted logistic regression models for all periods combined including period as a stratification variable in the models. Models were adjusted for (1) age, sex and ethnicity, and additionally for (2) demographic factors (marital and retirement status), (3) health behaviours (smoking habit, alcohol consumption and physical activity), adiposity (BMI) and other physiological risk factors (SBP and serum cholesterol), (4) prevalent disease (long-standing illness, CHD, stroke and diabetes) and (5) all of these covariates. Attenuation of the low versus high employment grade difference in recovery rate was calculated by comparing the coefficient from an adjusted model with that from the age, sex and ethnicity-adjusted model on the log odds scale. Sensitivity analyses were conducted using an alternative definition of recovery from poor physical and mental health: improvement in PCS/MCS ≥8 points. Analyses were performed using Stata V.13.1.

## Results

**Figure 1:**
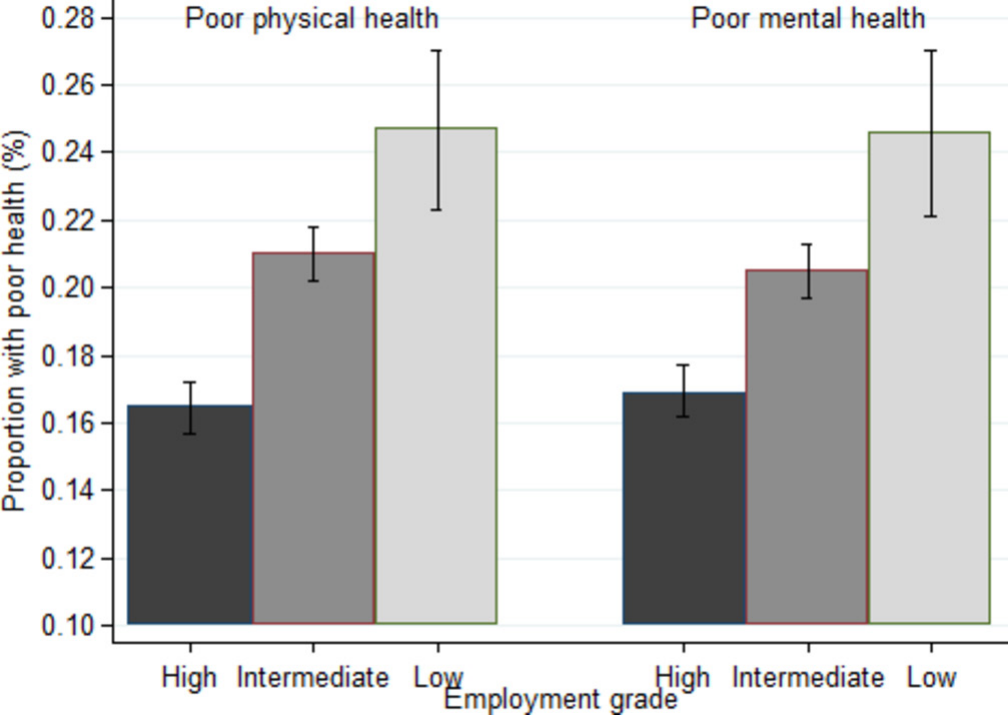
Proportion with 95% CI of participants with poor physical or mental health at the start of each period by employment grade, adjusted for age and sex. Test for trend: poor physical health, P<0.001; poor mental health, P<0.001.

**Figure 2:**
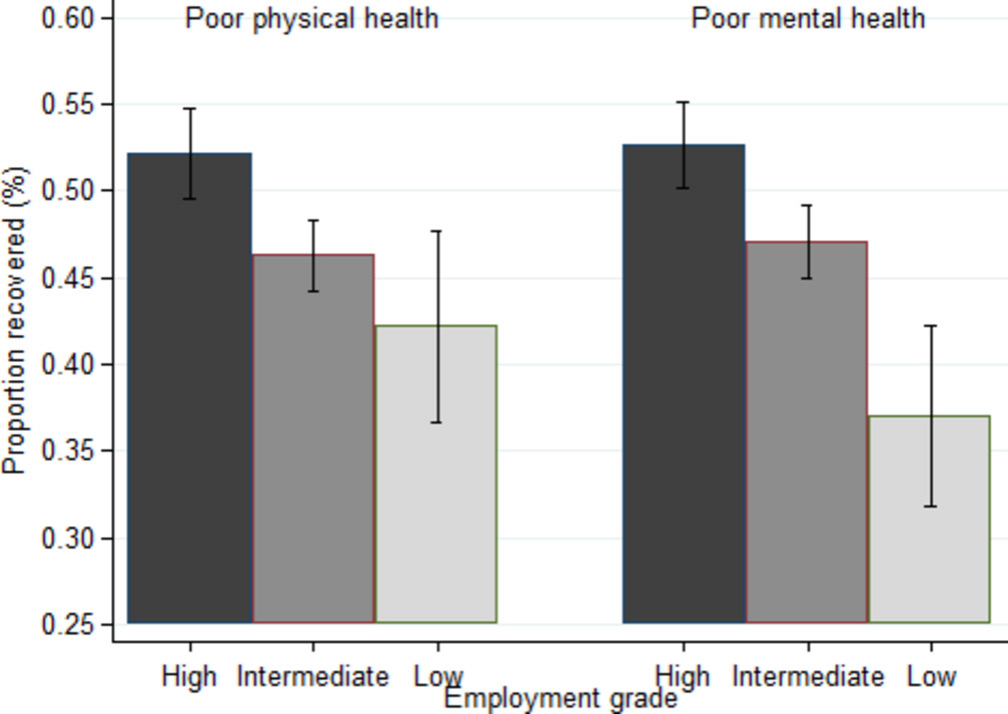
Proportion who recover with 95% CI, by employment grade, of participants with poor physical or mental health at the start of each period, adjusted for age and sex. Test for trend: poor physical health, P=0.004; poor mental health, P<0.001.

Of 24 001 person-observations, 4699 (19.6%) identified poor physical health and 4584 (19.1%) poor mental health at the beginning of the four observation periods, with 1178 (4.9%) identifying both conditions. There were socioeconomic gradients in the prevalence proportions of poor physical and mental health according to employment grade (figure 1). Approximately half the person-observations with poor health at the beginning of a period involved recovery (MCS 43%–52%, PCS 44%–50%) (table 1). The distribution of demographic factors, health behaviours and health status in those with poor physical or mental health at the beginning of each period is shown in online supplementary table S3. Participants in lower employment grades tended to have lower mean MCS and PCS at the beginning of each period and were less likely to recover from poor physical or mental health by the end of the period (online supplementary table S4). Trends in recovery by employment grade were seen in both men and women (data not shown). Combining the data across all four periods showed overall that the age and sex-adjusted odds of recovery from poor mental and physical health were lower in the lower employment grades (figure 2). Models differing in covariate adjustment showed that marital and retirement status and chronic disease accounted for little of the socioeconomic gradients in recovery. Health behaviours, adiposity, SBP and serum cholesterol explained half the gradient in recovery from poor physical health, less for poor mental health (table 2, 48% vs 28% (attenuations not shown)).

**Table 2:** Association of employment grade with recovery from poor physical and mental health across all periods

	Person-observations	Score, mean (SD)	Recovered, N (rate*)	OR (95% CI)				
				Adjusted for age, sex and ethnicity	Adjusted for all demographic factors†	Adjusted for age, sex, ethnicity, behaviours, BMI, risk factors‡	Adjusted for age, sex, ethnicity and prevalent disease§	Multiply adjusted¶
Poor physical health (n=4699)								
Employment grade								
High	1872	39.3 (7.1)	965 (51.6)	Ref	Ref	Ref	Ref	Ref
Intermediate	2193	37.4 (8.3)	1014 (46.2)	0.86 (0.75 to 0.98)	0.89 (0.78 to 1.01)	0.90 (0.79 to 1.03)	0.85 (0.75 to 0.98)	0.93 (0.81 to 1.06)
Low	634	34.4 (8.1)	246 (38.8)	0.73 (0.59 to 0.91)	0.77 (0.62 to 0.96)	0.85 (0.68 to 1.07)	0.71 (0.57 to 0.89)	0.85 (0.68 to 1.07)
P value for trend				0.002	0.013	0.086	0.001	0.142
Poor mental health (n=4584)								
Employment grade								
High	1896	38.8 (8.1)	955 (50.4)	Ref	Ref	Ref	Ref	Ref
Intermediate	2142	36.9 (8.5)	1014 (47.3)	0.84 (0.74 to 0.96)	0.89 (0.78 to 1.02)	0.88 (0.77 to 1.00)	0.85 (0.74 to 0.96)	0.93 (0.81 to 1.06)
Low	546	35.6 (8.2)	234 (42.9)	0.62 (0.50 to 0.78)	0.67 (0.54 to 0.84)	0.71 (0.57 to 0.89)	0.63 (0.51 to 0.78)	0.74 (0.59 to 0.93)
P value for trend				<0.001	0.001	0.002	<0.001	0.025

Cut-point for recovery depends on age and sex.

*Rate of recovery per 100 persons.

†Adjusted for age, sex, ethnicity, marital status and retirement status.

‡Adjusted for age, sex, ethnicity, smoking habit, alcohol consumption, physical activity, BMI, systolic blood pressure and serum cholesterol.

§Adjusted for age, sex, ethnicity, long-standing illness, CHD, stroke and diabetes.

¶Multiply adjusted for age, sex, marital status, ethnicity, retirement status, health behaviours (smoking habit, alcohol consumption and physical activity), BMI, systolic blood pressure, serum cholesterol and prevalent disease (long-standing illness, CHD, stroke and diabetes). Missing values imputed using multiple imputation with 20 imputed datasets.

BMI, body mass index; CHD, coronary heart disease.

The association of covariates with recovery from poor physical and mental health was examined in minimally and multiply adjusted models (online supplementary table S5). In the multiply adjusted model, low odds of recovery from poor physical health were observed in smokers, overweight and obese groups, and those either with long-standing illness or CHD. Fewer covariates were associated with recovery from poor mental health than with poor physical health (online supplementary table S6). Odds of recovery from poor mental health were reduced among those with long-standing illness and CHD.

In order to check that employment grade-recovery effects were not biased by systematic differences in PCS and MCS (severity) at the start of each observation period, we performed a sensitivity analysis in which recovery was defined uniformly as improvement in PCS or MCS of ≥8 during each period (online supplementary table S7). This alternative mode of analysis produced similar findings compared with those based on attaining a score above the lowest quintile (table 2).

## Discussion

### Main findings

Odds of recovery from poor mental and physical health were lower in lower employment grades. Socioeconomic inequality in recovery on the physical health component was accounted for partly by distribution of health behaviours, adiposity, SBP and serum cholesterol across the strata. Inequality in recovery on the mental component was less well explained by these potential intermediate factors. There was a tendency for those in the lower strata to have poorer health at the start of each round of observation, and accordingly a reduced likelihood of moving out of the bottom 20% of scores. Nevertheless, the socioeconomic gradients in odds of recovery from poor mental or poor physical health were independent of the respective initial SF-36 score.

This study evaluated whether socioeconomic status was a determinant of recovery both from poor mental and physical health among adults initially aged 39–63 years. Participants were followed up for 23 years with repeat collections of the SF-36 questionnaire and other information on health status at 5-year intervals (1991–2013). The socioeconomic gradient in recovery was similar across periods; therefore, the four periods of observation were combined with a multilevel logistic regression model. This method estimates error variances allowing for multiple observations on the same individual. As well as controlling for marital status and other personal characteristics, we were able to adjust for prevalent chronic disease and for health behaviours, adiposity, SBP and serum cholesterol at the start of each person-observation. Socioeconomic inequalities in the odds of recovery from poor physical and mental health were each partially explained by health behaviours and other physiological risk factors, as is the case with decline in health-related functioning, and incidence of cardiometabolic disease.^18 21^

Dropout rates were higher in the lower employment grades than higher, and this was a potential limitation. However, it is likely those who dropped out had poorer health and functioning than those who remained in the study, and estimates of the gradient in odds of recovery by employment grade are probably underestimated.

The SF-36 is a standardised method to assess physical and mental health where both scores have a mean value of 50 and an SD of 10 in the general US population.^17^ The PCS in particular is strongly associated with age and data are analysed over >20 years of follow-up, such that few younger participants would fall into the lowest quintile if a single cut-point defined poor health. Therefore, this study defined cut-points for poor health as the age–sex-specific lowest quintile of functioning scores at each measurement phase. The age–sex-specific lowest quintile for physical functioning was between 22 and 50 (online supplementary table S1) while for mental functioning it was between 40 and 51 (online supplementary table S2).

### Strengths and weaknesses in relation to other studies

Previous findings on socioeconomic differences in recovery from poor mental or physical health have been mixed, at least in part due to their small size. Socioeconomic status was not associated with recovery from a mobility limitation in a comparison of 185 non-manual and 447 manual men.^11^ Eighteen-month follow-up for recurrent or persistent common mental disorder among 750 adults in the UK Psychiatric Morbidity Survey found increased odds of caseness in the economically inactive group, but unemployed adults and those in lower strata of education or socioeconomic class had no significant disadvantage after controlling for baseline mental health (The Clinical Interview Schedule-Revised score).^22^ In contrast, higher socioeconomic status was associated with return to work and lower recurrence following psychiatric work disability in a larger study of almost 4000 adults.^7^ The present study, although based only on civil servants, has an effective sample size of >8000 as a result of combining four cycles of observation over the follow-up period.

### What the study adds

Some previous cohort studies based on education level suggest more rapid recovery from mobility disability is associated with years of school and college, with the implication that self-efficacy contributes importantly to the recovery aspect of socioeconomic inequality.^10 13^ The present study uses the SF-36, well characterised for measuring functional health status change and health inequality in the general population.^16 18 23^ Employment grade rather than education level here predicted recovery from poor physical and mental health. This measure of adult socioeconomic status characterises classes of individuals with similar income, pension rights, job security and work skills, all of which are resources that may directly or indirectly influence rate of recovery from poor health. Further, low employment grade is associated with clustering of adverse behavioural and vascular risk factors,^24 25^ and with rapid arterial ageing.^26^

The SF-36 questionnaire measures the functional aspects of health. Here, the socioeconomic gradient in the recovery from a low PCS was no longer significant in the maximally adjusted model, indicating that health behaviours, BMI, SBP and serum cholesterol together provide a good statistical explanation for the socioeconomic gradient. Although the association was halved in size in the maximally adjusted model, the same set of covariates had a weaker attenuating effect on inequality in recovery from poor self-rated mental health. We did not explore the extent to which each covariate accounted for the respective gradients since causal explanations were outside the scope of this analysis. It would appear that a common cause explanation for the physical and mental health gradients may be important.^27^ Adverse health-related behaviours and degree of adiposity were associated with lower socioeconomic status, and our analysis suggests they contributed to the gradient in both recovery outcomes.^28 29^

In conclusion, socioeconomic inequalities in the odds of recovery from poor physical and mental health, measured by multiple repeat data on the self-reported SF-36 instrument, were demonstrated over >20 years of follow-up. We adjusted for severity of ill-health at the start of each wave. The study sheds fresh light on the nature of health inequality, in showing that recovery from, as well as incidence of, poor health needs to receive detailed policy attention.

**What is already known on this subject**

- Socioeconomic gradients in incidence and prevalence of many diseases and adverse health states have been reported in many populations.
- In contrast, the role of socioeconomic status in recovery from poor physical and mental health is poorly documented.

**What this study adds**

- Physical and mental health was studied systematically using five waves of data collection over a 23-year period using the Short-Form 36 medical outcomes questionnaire.
- Participants in the higher employment grades with poor physical or mental health at the start of each period were more likely to have recovered after 5 years.
- The socioeconomic gradient in recovery was most striking for poor mental health—some 53% in the highest grade had recovered at 5 years, whereas the corresponding proportion in the lowest grade was 37%.
- The socioeconomic gradient in the time period spent with low functioning has implications for healthcare and other institutions in relation to return to work and domestic caregiving.

**Policy implications**

- The socioeconomic gradient in recovery from poor health, and the time period spent in states of low health-related functioning, has implications for health and social care provision, and employers, in relation to return to work and domestic caregiving.
- Recovery from mental health problems appears particularly to be affected by socioeconomic status, and targeted policy actions could produce considerable health gain.
- Our findings draw attention to the recovery period as a potential target for policies aiming to reduce health inequality.
