# Supplementary material for: Socioeconomic inequality in recovery from poor physical and mental health in mid-life and early old age: prospective Whitehall II cohort study
Source: J Epidemiol Community Health. 2018 Feb 8;72(4):309–13. doi: 10.1136/jech-2017-209584 (PMC5868522; doi:10.1136/jech-2017-209584)
Supplement: Supplementary file 1 [file jech-2017-209584supp001.pdf]

Socioeconomic inequality in recovery from poor physical and mental health in mid-life and early old age: prospective Whitehall II cohort study

Akihiro Tanaka, Martin J Shipley, Catherine A Welch, Nora Groce, Michael G Marmot, Mika Kivimaki, Archana Singh-Manoux, Eric J Brunner

SUPPLEMENTARY TABLES S1-S7

Supplementary Table S1: Lowest physical component score (PCS) quintile by age, sex and phase

Sex	Age group	Phase 3 (N=7,358)		Phase 5 (N=6,891)		Phase 7 (N=6,581)		Phase 9 (N=6,371)		Phase 11 (N=5,705)		Average Lowest PCS quintile
		N	Lowest PCS quintile	N	Lowest PCS quintile	N	Lowest PCS quintile	N	Lowest PCS quintile	N	Lowest PCS quintile	
Male	39-44	1,397	50.4	-	-	-	-	-	-	-	-	50.4
	45-49	1,435	49.9	1,023	48.9	-	-	-	-	-	-	49.4
	50-54	1,017	49.1	1,448	48.6	863	46.7	-	-	-	-	48.1
	55-59	1,048	47.8	1,016	47.2	1,447	46.2	869	47.0	-	-	47.1
	60-64	247	49.1	1,018	45.9	990	44.8	1,463	46.4	1,030	46.9	46.6
	65-69	-	-	381	47.2	931	42.4	948	44.2	1,241	44.0	44.5
	70-74	-	-	-	-	442	41.3	884	39.9	843	41.6	40.9
	75-79	-	-	-	-	-	-	391	38.5	742	36.9	37.7
	80-83	-	-	-	-	-	-	-	-	205	34.4	34.4
Female	39-44	517	48.1	-	-	-	-	-	-	-	-	48.1
	45-49	571	44.2	380	45.5	-	-	-	-	-	-	44.9
	50-54	471	43.0	533	42.6	346	43.3	-	-	-	-	43.0
	55-59	522	42.3	448	38.3	505	39.6	348	43.2	-	-	40.9
	60-64	133	40.8	472	39.3	409	36.5	510	41.1	409	42.8	40.1
	65-69	-	-	172	38.9	446	35.2	392	37.4	457	37.9	37.4
	70-74	-	-	-	-	202	29.6	386	32.2	350	33.2	31.7
	75-79	-	-	-	-	-	-	180	30.0	328	28.2	29.1
	80-83	-	-	-	-	-	-	-	-	100	22.3	22.3

Supplementary Table S2: Lowest mental component score (MCS) quintile by age, sex and phase

Sex	Age group	Phase 3 (N=7,358)		Phase 5 (N=6,891)		Phase 7 (N=6,581)		Phase 9 (N=6,371)		Phase 11 (N=5,705)		Average Lowest MCS quintile
		N	Lowest MCS quintile	N	Lowest MCS quintile	N	Lowest MCS quintile	N	Lowest MCS quintile	N	Lowest MCS quintile	
Male	39-44	1,397	44.9	-	-	-	-	-	-	-		44.9
	45-49	1,435	45.4	1,023	41.9	-	-	-	-	-	-	43.7
	50-54	1,017	47.2	1,448	45.0	863	43.8	-	-	-	-	45.3
	55-59	1,048	48.7	1,016	48.1	1,447	46.5	869	47.6	-	-	47.7
	60-64	247	51.9	1,018	50.3	990	49.3	1,463	50.0	1,030	49.3	50.2
	65-69	-	-	381	51.2	931	50.7	948	51.2	1,241	50.7	51.0
	70-74	-	-	-	-	442	50.7	884	51.1	843	50.2	50.7
	75-79	-	-	-	-	-	-	391	49.6	742	50.0	49.8
	80-83	-	-	-	-	-	-	-	-	205	49.1	49.1
Female	39-44	517	42.3	-	-	-	-	-	-	-	-	42.3
	45-49	571	41.9	380	37.4	-	-	-	-	-	-	39.7
	50-54	471	45.9	533	38.6	346	42.8	-	-	-	-	42.4
	55-59	522	46.8	448	42.4	505	42.9	348	44.4	-	-	44.1
	60-64	133	46.6	472	46.9	409	46.1	510	47.7	409	46.4	46.7
	65-69	-	-	172	42.9	446	47.2	392	48.8	457	45.7	46.2
	70-74	-	-	-	-	202	46.1	386	47.3	350	46.4	46.6
	75-79	-	-	-	-	-	-	180	45.7	328	44.3	45.0
	80-83	-	-	-	-	-	-	-	-	100	47.4	47.4

Supplementary Table S3: Distribution of risk factors at start of each period among the participants with either low physical or low mental health functioning

		Period 1 (N=2,064)	Period 2 (N=2,053)	Period 3 (N=2,156)	Period 4 (N=1,832)
**Demographic factors**					
Age, mean (SD)		49.6 (6.0)	55.3 (6.0)	60.6 (5.8)	65.5 (5.8)
Sex, N (%)	Male	1,448 (70.2)	1,461 (71.2)	1,578 (73.2)	1,359 (74.2)
	Female	616 (29.8)	592 (28.8)	578 (26.8)	473 (25.8)
Ethnicity, N (%)	White	1,859 (90.1)	1,850 (90.1)	1,972 (91.5)	1,666 (90.9)
	South Asian	137 (6.6)	142 (6.9)	122 (5.7)	111 (6.1)
	Black	50 (2.4)	43 (2.1)	44 (2.0)	39 (2.1)
	Other	16 (0.8)	17 (0.8)	14 (0.6)	15 (0.8)
	Missing[a]	*2 (0.1)*	*1 (0.0)*	*4 (0.2)*	*1 (0.1)*
Marital status, N (%)	Married/cohabiting	1,527 (74.0)	1,508 (73.5)	1,577 (73.1)	1,313 (71.7)
	Single	338 (16.4)	243 (11.8)	292 (13.5)	232 (12.7)
	Divorced/widowed	197 (9.5)	217 (10.6)	285 (13.2)	257 (14.0)
	Missing	*2 (0.1)*	*85 (4.1)*	*2 (0.1)*	*30 (1.6)*
Employment grade, N (%)	High	761 (36.9)	825 (40.2)	976 (45.3)	796 (43.4)
	Intermediate	1,002 (48.5)	965 (47.0)	965 (44.8)	836 (45.6)
	Low	301 (14.6)	263 (12.8)	215 (10.0)	200 (10.9)
Retirement status, N (%)	Not retired	1,899 (92.0)	1,507 (73.4)	1,220 (56.6)	654 (35.7)
	Retired	165 (8.0)	546 (26.6)	936 (43.4)	1,178 (64.3)
**Health behaviours**					
Smoking habit, N (%)	Never	858 (41.6)	927 (45.2)	1,004 (46.6)	750 (40.9)
	Ex-smoker	766 (37.1)	875 (42.6)	957 (44.4)	872 (47.6)
	Current smoker	336 (16.3)	235 (11.4)	187 (8.7)	123 (6.7)
	Missing	*104 (5.0)*	*16 (0.8)*	*8 (0.4)*	*87 (4.7)*
Alcohol consumption, N (%)	None	438 (21.2)	380 (18.5)	400 (18.6)	405 (22.1)
	Moderate	1,297 (62.8)	1,156 (56.3)	1,320 (61.2)	1,076 (58.7)
	Heavy	327 (15.8)	485 (23.6)	419 (19.4)	317 (17.3)
	Missing	*2 (0.1)*	*32 (1.6)*	*17 (0.8)*	*34 (1.9)*
Physical activity, N (%)	Sufficiently active	931 (45.1)	940 (45.8)	1125 (52.2)	919 (50.2)
	Moderately active	636 (30.8)	436 (21.2)	443 (20.5)	392 (21.4)
	Inactive	497 (24.1)	657 (32.0)	578 (26.8)	511 (27.9)
	Missing	*0 (0.0)*	*20 (1.0)*	*10 (0.5)*	*10 (0.5)*
**Disease and health indicators**					
Body mass index, N (%)	Normal	996 (48.3)	670 (32.6)	696 (32.3)	581 (31.7)
	Overweight	742 (35.9)	693 (33.8)	874 (40.5)	726 (39.6)
	Obese	219 (10.6)	285 (13.9)	473 (21.9)	404 (22.1)
	Missing	*107 (5.2)*	*405 (19.7)*	*113 (5.2)*	*121 (6.6)*
Systolic blood pressure (mmHg), mean (SD)		119.8 (13.3)	122.1 (15.9)	127.5 (16.6)	125.3 (16.1)
	Missing	*102 (5.2)*	*192 (10.3)*	*101 (4.9)*	*117 (6.8)*
Total cholesterol (mmol l^{-1}), mean (SD)		6.4 (1.1)	5.9 (1.1)	5.7 (1.0)	5.1 (1.1)
	Missing	*114 (5.8)*	*207 (11.2)*	*139 (6.9)*	*163 (9.8)*
Longstanding illness, N (%)	No	1,015 (49.2)	679 (33.1)	564 (26.2)	391 (21.3)
	Yes	1,049 (50.8)	1,373 (66.9)	1,590 (73.7)	1,425 (77.8)
	Missing	*0 (0.0)*	*1 (0.0)*	*2 (0.1)*	*16 (0.9)*
CHD, N (%)	No	1,800 (87.2)	1,633 (79.5)	1,645 (76.3)	1,266 (69.1)
	Yes	264 (12.8)	420 (20.5)	511 (23.7)	566 (30.9)
Stroke, N (%)	No	2061 (99.9)	2,046 (99.7)	2,137 (99.1)	1,797 (98.1)
	Yes	3 (0.1)	7 (0.3)	19 (0.9)	35 (1.9)
Diabetes, N (%)	No	1,818 (88.1)	1,705 (83.1)	1,803 (83.6)	1,408 (76.9)
	Yes	54 (2.6)	113 (5.5)	187 (8.7)	258 (14.1)
	Missing	*192 (9.3)*	*235 (11.4)*	*166 (7.7)*	*166 (9.1)*

[a] In the main analysis, participants with missing ethnicity are assumed to be white

Supplementary Table S4: Odds of recovery from poor physical and mental health by employment grade for each period

	Period 1			Period 2			Period 3			Period 4		
	Score, mean (SD)	Recovered / N (%)	OR[a] (95% CI)	Score, mean (SD)	Recovered / N (%)	OR (95% CI)	Score, mean (SD)	Recovered /N (%)	OR (95% CI)	Score, mean (SD)	Recovered /N (%)	OR (95% CI)
**Low Physical Health N=(1,186/1,145/1,302/1,066)**												
Employment grade												
High	43.0 (5.9)	221/413 (53.5)	Ref	40.6 (6.8)	226/452 (50.0)	Ref	37.9 (7.1)	305/554 (55.1)	Ref	36.4 (6.8)	213/453 (47.0)	Ref
Interme-diate	41.1 (7.2)	275/566 (48.6)	0.83 (0.64, 1.08)	38.0 (8.0)	229/538 (42.6)	0.77 (0.59, 0.99)	36.1 (8.2)	288/604 (47.7)	0.71 (0.56, 0.90)	34.1 (8.0)	222/485 (45.8)	0.97 (0.75, 1.67)
Low	37.2 (7.7)	84/207 (40.6)	0.64 (0.44, 0.93)	34.5 (7.5)	49/155 (31.6)	0.52 (0.34, 0.80)	32.9 (8.3)	56/144 (38.9)	0.45 (0.29, 0.68)	31.3 (8.0)	57/128 (44.5)	1.02 (0.97, 1.01)
P-value for trend			0.020			0.002			<0.001			0.975
**Low Mental Health N=(1,096/1,201/1,217/1,070)**												
Employment grade												
High	37.1 (7.4)	217/424 (51.2)	Ref	37.0 (8.3)	229/454 (50.4)	Ref	39.7 (8.3)	309/562 (55.0)	Ref	41.0 (7.8)	200/456 (43.9)	Ref
Interme-diate	35.0 (7.8)	246/536 (45.9)	0.78 (0.60, 1.01)	35.4 (8.4)	282/578 (48.8)	0.86 (0.67, 1.11)	37.7 (8.8)	267/534 (50.0)	0.74 (0.58, 0.95)	39.8 (8.2)	219/494 (44.3)	0.97 (0.75, 1.26)
Low	34.4 (8.1)	53/136 (39.0)	0.53 (0.34, 0.82)	34.4 (8.2)	86/169 (50.9)	0.82 (0.56, 1.21)	37.1 (8.0)	54/121 (44.6)	0.54 (0.35, 0.82)	37.3 (8.3)	41/120 (34.2)	0.58 (0.37, 0.90)
P-value for trend			0.003			0.225			0.001			0.063

[a] Odds ratios are adjusted for age and sex

Supplementary Table S5: Association of covariates with recovery from poor physical health across all periods

		Person observations	Recovered, N (Rate[a])	OR (95% CI) Adjusted for age, sex[b] and ethnicity	OR (95% CI) Maximally adjusted[c]
Age group	39-44	334	181 (54.2)	1.30 (1.01, 1.66)	0.92 (0.71, 1.20)
	45-49	585	288 (49.2)	1.07 (0.87, 1.31)	0.89 (0.72, 1.10)
	50-54	810	377 (46.5)	0.97 (0.81, 1.17)	0.88 (0.72, 1.06)
	55-59	1,093	516 (47.2)	Ref	Ref
	60-64	947	438 (46.3)	0.96 (0.81, 1.14)	1.09 (0.90, 1.33)
	65-69	575	262 (45.6)	0.93 (0.76, 1.14)	1.14 (0.90, 1.44)
	70+	355	163 (45.9)	0.94 (0.74, 1.20)	1.29 (0.97, 1.70)
Sex	Male	3,355	1,637 (48.8)	Ref	Ref
	Female	1,344	588 (43.8)	0.88 (0.77, 1.00)	1.15 (0.99, 1.35)
Ethnicity	White	4,162	2055 (49.4)	Ref	Ref
	South Asian	361	111 (30.7)	0.47 (0.37, 0.59)	0.45 (0.35, 0.58)
	Black	134	48 (35.8)	0.60 (0.42, 0.86)	0.67 (0.45, 0.98)
	Other	42	11 (26.2)	0.37 (0.19, 0.75)	0.37 (0.18, 0.75)
Marital status	Married/cohabiting	3,538	1,746 (49.3)	Ref	Ref
	Single	618	243 (39.3)	0.65 (0.54, 0.78)	0.69 (0.57, 0.83)
	Divorced/widowed	484	216 (44.6)	0.86 (0.71, 1.05)	0.92 (0.75, 1.13)
	Missing	*59*	*20 (33.9)*	-	-
Retirement status	Not retired	2,918	1,428 (48.9)	Ref	Ref
	Retired	1,781	797 (44.8)	0.81 (0.69, 0.95)	0.82 (0.69, 0.97)
Smoking habit	Never	2,039	999 (49.0)	Ref	Ref
	Ex-smoker	2,034	961 (47.2)	0.97 (0.85, 1.10)	0.91 (0.80, 1.04)
	Current smoker	522	217 (41.6)	0.73 (0.60, 0.90)	0.68 (0.55, 0.84)
	Missing	*104*	*48 (46.2)*	-	-
Alcohol consumption	None	1,063	433 (40.7)	Ref	Ref
	Moderate	2,744	1,340 (48.8)	1.23 (1.06, 1.43)	1.10 (0.94, 1.30)
	Heavy	843	427 (50.7)	1.26 (1.04, 1.53)	1.28 (1.04, 1.57)
	Missing	*49*	*25 (51)*	-	-
Physical activity	Sufficiently active	2,163	1,121 (51.8)	Ref	Ref
	Moderately active	1,089	501 (46.0)	0.81 (0.70, 0.94)	0.93 (0.77, 1.13)
	Inactive	1,421	593 (41.7)	0.73 (0.63, 0.84)	0.92 (0.77, 1.10)
	Missing	*26*	*10 (38.5)*	-	-
BMI	Normal	1,516	805 (53.1)	Ref	Ref
	Overweight	1,758	866 (49.3)	0.85 (0.74, 0.98)	0.82 (0.72, 0.95)
	Obese	977	364 (37.3)	0.52 (0.44, 0.61)	0.53 (0.44, 0.63)
	Missing	448	190 (42.4)	-	-
Systolic blood pressure (100mmHg)		-	-	0.93 (0.79, 1.10)	1.05 (0.88, 1.26)
Total cholesterol (mmol l^{-1})		-	-	1.02 (0.96, 1.07)	0.97 (0.92, 1.03)
Longstanding Illness	No	1,072	653 (60.9)	Ref	Ref
	Yes	3,615	1,565 (43.3)	0.49 (0.42, 0.57)	0.51 (0.44, 0.59)
	Missing	*12*	*7 (58.3)*	-	-
CHD	No	3,460	1,753 (50.7)	Ref	Ref
	Yes	1,239	472 (38.1)	0.62 (0.54, 0.71)	0.69 (0.60, 0.79)
Stroke	No	4,646	2,203 (47.4)	Ref	Ref
	Yes	53	22 (41.5)	0.83 (0.48, 1.45)	0.99 (0.56, 1.75)
Diabetes	No	3,814	1,870 (49.0)	Ref	Ref
	Yes	428	166 (38.8)	0.72 (0.58, 0.89)	0.91 (0.73, 1.13)
	Missing	*457*	*189 (41.4)*	-	-

[a] Rate of recovery per 100 persons

[b] Age group, sex and ethnicity are adjusted for each other

[c] Multiply adjusted for: age, sex, marital status, ethnicity, employment grade, retirement status, health behaviours (smoking habit, alcohol consumption and physical activity), BMI, systolic blood pressure, total cholesterol and prevalent disease (longstanding illness, CHD, stroke and diabetes)

Supplementary Table S6: Association of covariates with recovery from poor mental health across all periods

		Person observations	Recovered N (Rate[a])	OR (95% CI) Adjusted for age, sex[b] and ethnicity	OR (95% CI) Maximally adjusted[c]
Age group	39-44	328	148 (45.1)	0.84 (0.65, 1.08)	0.75 (0.58, 0.98)
	45-49	582	303 (52.1)	1.11 (0.91, 1.36)	1.05 (0.85, 1.30)
	50-54	792	383 (48.4)	0.96 (0.80, 1.15)	0.93 (0.77, 1.12)
	55-59	1,071	528 (49.3)	Ref)	Ref
	60-64	903	426 (47.2)	0.92 (0.77, 1.10)	0.98 (0.81, 1.19)
	65-69	528	243 (46.0)	0.88 (0.72, 1.09)	0.96 (0.76, 1.23)
	70+	380	172 (45.3)	0.87 (0.68, 1.10)	0.99 (0.75, 1.30)
Sex	Male	3,328	1,552 (46.6)	Ref	Ref
	Female	1,256	651 (51.8)	1.24 (1.09, 1.41)	1.52 (1.31, 1.77)
Ethnicity	White	4,190	2,032 (48.5)	Ref	Ref
	South Asian	284	111 (39.1)	0.68 (0.53, 0.87)	0.79 (0.61, 1.04)
	Black	74	44 (59.5)	1.48 (0.93, 2.38)	1.71 (1.05, 2.79)
	Other	36	16 (44.4)	0.81 (0.42, 1.57)	0.92 (0.47, 1.81)
Marital status	Married/cohabiting	3,190	1,590 (49.8)	Ref	Ref
	Single	695	268 (38.6)	0.59 (0.50, 0.70)	0.64 (0.54, 0.77)
	Divorced/widowed	619	312 (50.4)	0.96 (0.81, 1.15)	1.03 (0.86, 1.24)
	Missing	*80*	*33 (41.3)*	-	-
Retirement Status	Not retired	3,067	1,510 (49.2)	Ref	Ref
	Retired	1,517	693 (45.7)	0.87 (0.73, 1.04)	0.92 (0.77, 1.10)
Smoking habit	Never	1,996	989 (49.5)	Ref	Ref
	Ex-smoker	1,955	920 (47.1)	0.91 (0.80, 1.04)	0.90 (0.79, 1.03)
	Current smoker	493	232 (47.1)	0.88 (0.72, 1.08)	0.93 (0.76, 1.14)
	Missing	*140*	*62 (44.3)*	-	-
Alcohol consumption	None	878	344 (39.2)	Ref	Ref
	Moderate	2,724	1,385 (50.8)	1.68 (1.43, 1.98)	1.52 (1.29, 1.79)
	Heavy	927	444 (47.9)	1.52 (1.25, 1.84)	1.39 (1.14, 1.71)
	Missing	*55*	*30 (54.5)*	-	-
Physical Activity	Sufficiently active	2,202	1,106 (50.2)	Ref	Ref
	Moderately active	1,084	534 (49.3)	0.95 (0.82, 1.10)	1.01 (0.84, 1.23)
	Inactive	1,275	550 (43.1)	0.72 (0.62, 0.83)	0.83 (0.68, 1.01)
	Missing	*23*	*13 (56.5)*	-	-
BMI	Normal	1,825	882 (48.3)	Ref	Ref
	Overweight	1,676	818 (48.8)	1.03 (0.9, 1.18)	1.06 (0.92, 1.22)
	Obese	650	303 (46.6)	0.9 (0.75, 1.08)	1.03 (0.86, 1.25)
	Missing	*433*	*200 (46.2)*	-	-
Systolic blood pressure (100mmHg)		-	-	0.91 (0.77, 1.08)	0.94 (0.79, 1.13)
Total cholesterol (mmol l^{-1})		-	-	0.99 (0.94, 1.05)	0.98 (0.93, 1.04)
Longstanding Illness	No	1,747	916 (52.4)	Ref	Ref
	Yes	2,826	1,281 (45.3)	0.75 (0.66, 0.85)	0.78 (0.69, 0.89)
	Missing	*11*	*6 (54.5)*	-	-
CHD	No	3,662	1,806 (49.3)	Ref	Ref
	Yes	922	397 (43.1)	0.79 (0.68, 0.91)	0.82 (0.70, 0.96)
Stroke	No	4,557	2,189 (48.0)	Ref	Ref
	Yes	27	14 (51.9)	1.32 (0.62, 2.84)	1.57 (0.72, 3.40)
Diabetes	No	3,839	1,865 (48.6)	Ref	Ref
	Yes	303	146 (48.2)	1.06 (0.83, 1.35)	1.11 (0.86, 1.44)
	Missing	*442*	*192 (43.4)*	-	-

[a] Rate of recovery per 100 persons

[b] Age group, sex and ethnicity are adjusted for each other

[c] Multiply adjusted for: age, sex, marital status, ethnicity, employment grade, retirement status, health behaviours (smoking habit, alcohol consumption and physical activity), BMI, systolic blood pressure, total cholesterol and prevalent disease (longstanding illness, CHD, stroke and diabetes)

Supplementary Table S7: Association of employment grade with recovery from poor physical and mental health across all periods – recover if score difference (end of period – start of period) ≥ 8

	Person observations	Score, mean (SD)	Recovered, N (Rate[a])	OR (95% CI)				
				Adjusted for age, sex and ethnicity	Adjusted for all demographic factors[b]	Adjusted for age, sex, ethnicity and health behaviours[c]	Adjusted for age, sex, ethnicity and prevalent disease[d]	Multiply adjusted[e]
Low Physical Health (N=4,699)								
Employment grade								
High	1,872	39.3 (7.1)	617 (33.0)	Ref	Ref	Ref	Ref	Ref
Intermediate	2,193	37.4 (8.3)	679 (31.0)	0.90 (0.78, 1.03)	0.92 (0.80, 1.06)	0.92 (0.80, 1.06)	0.90 (0.79, 1.04)	0.95 (0.82, 1.09)
Low	634	34.4 (8.1)	163 (25.7)	0.67 (0.53, 0.85)	0.71 (0.56, 0.90)	0.73 (0.57, 0.94)	0.67 (0.53, 0.85)	0.75 (0.59, 0.97)
P-value for trend				0.002	0.011	0.023	0.002	0.056
Low Mental Health (N=4,584)								
Employment grade								
High	1,896	38.8 (8.1)	927 (48.9)	Ref	Ref	Ref	Ref	Ref
Intermediate	2,142	36.9 (8.5)	1,058 (48.9)	0.92 (0.81, 1.05)	0.96 (0.84, 1.09)	0.93 (0.82, 1.07)	0.93 (0.81, 1.06)	0.97 (0.85, 1.11)
Low	234	35.6 (8.2)	258 (47.3)	0.77 (0.62, 0.96)	0.80 (0.64, 1.00)	0.82 (0.66, 1.03)	0.78 (0.62, 0.97)	0.85 (0.68, 1.07)
P-value for trend				<0.001	0.087	0.089	0.032	0.222

[a] Rate of recovery per 100 persons

[b] Adjusted for: age, sex, ethnicity, marital status and retirement status.

[c] Adjusted for age, sex, ethnicity, smoking habit, alcohol consumption, physical activity and BMI

[d] Adjusted for: age, sex, ethnicity, longstanding illness, CHD, stroke and diabetes

[e] Multiply adjusted for: age, sex, marital status, ethnicity, retirement status, health behaviours (smoking habit, alcohol consumption and physical activity), BMI and prevalent disease (longstanding illness, CHD, stroke and diabetes). Missing values imputed using multiple imputation with 20 imputed datasets.
